# Usefulness of protocol-based pharmacotherapy management by pharmacists in cancer patients: a retrospective observational study

**DOI:** 10.1186/s40780-025-00504-8

**Published:** 2025-11-12

**Authors:** Satomi Sumikawa, Noriaki Hidaka, Yuya Sakamoto, Noboru Yamashita, Shinichi Watanabe, Mamoru Tanaka

**Affiliations:** 1 Division of Pharmacy, Ehime University Hospital, 454 Shitsukawa, Toon, Ehime 791-0295 Japan; 2 Department of Clinical Pharmacy, College of Pharmaceutical Sciences, Matsuyama University, 4-2 Bunkyo-Cho, Matsuyama, Ehime 790-8578 Japan

**Keywords:** PBPM, HBV reactivation, HBV-DNA, Anti-vascular endothelial growth factor therapy, Urine creatinine ratio, anti-EGFR antibody, Serum magnesium

## Abstract

**Background:**

A team-based approach is essential to provide cancer patients with high-quality treatment. To ensure the best possible care while reducing the workload of physicians, Ehime University Hospital has introduced three protocol-based pharmacotherapy management (PBPM) strategies in the field of chemotherapy. First, we introduced PBPM to avoid reactivation of hepatitis B virus (HBV) in patients receiving immunosuppressive therapy or chemotherapy. In this PBPM strategy, pharmacists added laboratory test orders for patients who require regular HBV-DNA quantification (HBV-PBPM). Second, we devised PBPM for measurement of the urine protein/creatinine ratio (UPC) in patients receiving anti-vascular endothelial growth factor therapy. Finally, we introduced PBPM for measurement of serum magnesium in patients receiving anti-epidermal growth factor receptor antibody therapy (Mg-PBPM). In this study, we evaluated the usefulness of these three PBPM strategies in outpatients receiving chemotherapy.

**Methods:**

The study included patients treated in the outpatient chemotherapy unit between July 2021 and February 2023. Rates of compliance with laboratory tests in the 6 months before and after introduction of PBPM were compared.

**Results:**

Compliance with HBV-DNA quantification improved significantly from 66.3% before PBPM to 86.7% after implementation of PBPM (*p* = 0.002). The median duration of noncompliance was significantly shorter after initiation of PBPM (*p* = 0.021). Compliance with measurement of UPC was already greater than 95% before PBPM and showed no change after implementation (98.7% pre-PBPM vs 99.3% post-PBPM). Compliance with measurement of serum magnesium improved from 95.8% pre-PBPM to 99.2% after starting PBPM, but the improvement was not statistically significant.

**Conclusions:**

Introduction of PBPM improves compliance with the laboratory tests required in cancer patients during chemotherapy and enables safer delivery of treatment.

## Background

Cancer treatment is becoming increasingly sophisticated and complex, necessitating a team-based approach to ensure that patients receive high-quality treatment. For an effective team-based approach, each healthcare professional must use their expertise, share objectives and information, and divide responsibilities. Collaborative and complementary roles among healthcare providers enable appropriate patient-centered care. In Japan, a 2010 directive from the Ministry of Health, Labour and Welfare recommended pharmacist participation as part of a team-based approach using protocols that were previously agreed upon [1]. These protocols allow pharmacists to make changes to type of medication, dosages, methods of administration, and testing on the basis of shared professional knowledge.

Since the introduction of the PBPM Implementation Manual by the Japanese Society of Pharmaceutical Health Care and Sciences in 2016 and practical examples provided by the Japanese Society of Hospital Pharmacists, an increasing number of institutions have initiated protocol-based pharmacotherapy management (PBPM) [2, 3]. Effective PBPM requires identification of any relevant problems at a facility and collaborative development of solutions by healthcare professionals. Pharmacist-led interventions can increase patient safety, particularly in cases where guidelines and package inserts are not being followed.

It has been reported that reactivation of hepatitis B virus (HBV) can be fatal in HBV carriers and in patients with a history of HBV infection who have received immunosuppressive therapy or cancer chemotherapy. Therefore, efforts to prevent reactivation are important in patients who require regular HBV-DNA quantitative testing [4–6]. Furthermore, laboratory tests should be performed at each treatment session to determine the feasibility of administering anticancer agents and to detect any adverse effects of these agents at an early stage. In this study, we reviewed the pharmacists’ queries regarding prescriptions received at our outpatient chemotherapy unit over a one-year period and introduced three PBPM strategies that were considered effective. The first PBPM strategy was designed to avoid reactivation of HBV by immunosuppressive therapy or chemotherapy (HBV-PBPM). The aim of the second strategy was to ensure that the urine protein/creatinine ratio (UPC) was measured in patients receiving anti-vascular endothelial growth factor (VEGF) therapy (UPC-PBPM). The third strategy was designed to ensure that serum magnesium was monitored in patients receiving anti-epidermal growth factor receptor (EGFR) antibody therapy (Mg-PBPM). In this study, we evaluated the usefulness of PBPM by investigating rates of compliance with laboratory tests before and after implementation of these strategies.

## Methods

### Development of effective PBPM

To identify useful PBPM strategies at our facility, we retrospectively reviewed the pharmacists’ queries regarding prescriptions between April 1, 2021 and March 31, 2022 by searching the electronic medical records and calculating the acceptance rate. The acceptance rate was calculated by dividing the number of changes proposed by the pharmacist that were accepted by physicians by the number of pharmacists’ queries made during the study period.

### Evaluation of the usefulness of the three PBPM strategies

#### HBV-PBPM

**Fig. 1:**
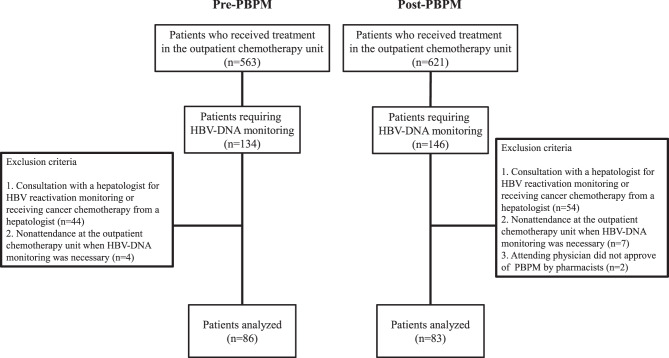
Selection of patients for HBV-related PBPM. After exclusions, 169 subjects were included in the analysis. HBV, hepatitis B virus; PBPM, protocol-based pharmacotherapy management

Of 1184 patients who received immunosuppressive therapy or chemotherapy in our outpatient chemotherapy unit between July 2021 and July 2022, 280 were HBsAg-positive and/or HBsAg/HBc antibody-positive. Among these 280 patients, those who met any of the following four criteria were excluded: consultation with a hepatologist for HBV reactivation monitoring; receiving cancer chemotherapy prescribed by a hepatologist; nonattendance at the outpatient chemotherapy unit when HBV-DNA monitoring was necessary; and having an attending physician who did not approve of PBPM by pharmacists. Patients who consulted a hepatologist were excluded from this study because they fall outside the scope of pharmacist-led PBPM. Finally, data for 169 patients were analyzed (Fig. 1). In accordance with the Hepatitis B Treatment Guidelines (4th edition) [4], compliance was evaluated as follows. Patients were assessed as being compliant with rituximab, obinutuzumab, and fludarabine if they underwent monthly testing (every 30 days) and as being compliant with immunosuppressive therapy (abatacept, infliximab, and tocilizumab) and other chemotherapy agents if testing was performed every 3 months (90 days). Other chemotherapies included single-agent or combination therapies, including immune checkpoint inhibitors (ICIs), cytotoxic agents, and molecular targeted therapies, excluding single-agent hormone therapies. PBPM was introduced in January 2022. The first month after its introduction was designated as a transition (operational awareness) period. The period between July and December 2021 was defined as before the start of PBPM and the period between February and July 2022 as after the start of PBPM.

#### UPC-PBPM

Patients who received anti-VEGF agents (bevacizumab, ramucirumab, aflibercept) between February 2022 and February 2023 were evaluated. After the exclusion of nine patients whose attending physicians did not approve of PBPM by pharmacists, a total of 1219 doses were analyzed. Compliance was defined as having the UPC ratio measured on the day of treatment. PBPM was introduced in August 2022. The first month after its introduction was designated as a transition (operational awareness) period. The period between February 2022 and July 2022 was designated as the period before introduction of PBPM and the period between September 2022 and February 2023 as the period after introduction of PBPM.

#### Mg-PBPM

Patients receiving anti-EGFR antibody therapy (cetuximab, panitumumab) between February 2022 and February 2023 were evaluated. During the study period, there were no cases in which authority was not delegated from physicians to pharmacists. A total of 217 doses were analyzed. Compliance was defined as having serum magnesium measured on the day of treatment. The first month after its introduction was designated as a transition (operational awareness) period. The period between February 2022 and July 2022 was designated as the period before introduction of PBPM, and the period between September 2022 and February 2023 as the period after introduction of PBPM.

### Changes in queries by pharmacists after introduction of PBPM and in the annual number of tests ordered by pharmacists

We investigated changes in the types of queries after introduction of the three types of PBPM. We assessed the number of queries and the number of cases accepted regarding laboratory test items for the 2021 fiscal year (FY) before introduction of PBPM, the 2022 FY during the PBPM introduction period, and the 2023 FY after introduction of PBPM. We also investigated the annual changes in the numbers of tests ordered by pharmacists for the three types of PBPM.

### Statistical analysis

Qualitative data were analyzed using Fisher’s exact test and quantitative data using the Mann‒Whitney *U* test. All statistical analyses were performed utilizing Easy-R (EZR) version 1.61 (2022; Jichi Medical University, Saitama, Japan) [7]. A P-value of < 0.05 was considered statistically significant.

## Results

### Development of effective PBPM

Between April 2021 and March 2022, 480 queries from pharmacist regarding prescriptions were recorded. The most common errors concerned an order (*n* = 116, acceptance rate 94.8%), followed by HBV-related issues (*n* = 94, acceptance rate 63.8%) and additional laboratory tests (*n* = 81, acceptance rate 85.2%). Before the start of PBPM, there were 480 queries regarding prescriptions in the outpatient chemotherapy unit, with an overall acceptance rate of 76.0% (Table 1). The prescription queries were HBV-related in about 20% of cases.

**Table 1:** Content and percentage of pharmacists’ queries regarding prescriptions and physicians’ acceptance rates

Nature of prescription query	Proposal (*n*)	Accepted (*n*)	Acceptance rate (%)
Order error ex. Not ordered, Differences between categories (inpatient/outpatient)	116	110	94.8
Hepatitis B-related issues	94	60	63.8
Additional laboratory tests	81	69	85.2
Additional zoledronic acid or denosumab	46	42	91.3
Change in anticancer drug dosage	43	24	55.8
Administer based on blood test results	39	10	25.6
Change in supportive care	34	28	82.4
Other	16	12	75.0
Adding or removing vitamin B12 preparations	11	10	90.9
Total	480	365	76.0

### Usefulness of the three PBPM strategies

#### HBV-PBPM

The final analysis included 86 patients before PBPM and 83 patients after PBPM. After the start of PBPM, the frequency of laboratory tests ordered by pharmacists increased significantly to 25% (*p* < 0.001). The median duration of noncompliance was 41 days before PBPM and 29 days after starting PBPM. The range of the noncompliance period was significantly shorter after initiation of PBPM (*p* = 0.021) (Table 2). HBV-DNA should have been measured at monthly intervals in patients receiving rituximab who were HBs/HBc antibody-positive, but were not measured in three cases during the study period. Two of these patients were from the pre-PBPM period and one was from the post-PBPM period. In the two patients who received rituximab before PBPM, the durations of non-compliance were 33 days and 41 days. One patient received rituximab after initiation of PBPM, and the duration of non-compliance was 65 days. The rate of compliance with HBV-DNA quantification improved significantly from 66.3% to 86.7% (*p* = 0.002) (Table 2).

**Table 2:** Comparison of rates of compliance with laboratory testing and patient background characteristics before and after HBV-related PBPM

Variable		Pre-PBPM	Post-PBPM	*P*-value
Patients (n)		86	83	
Male sex, n (%)		48 (56)	44 (53)	0.759
Median age (range), years		71 (26-88)	72 (27-90)	0.755
Department (n)	Hematology	21 (24.4)	25 (30.1)	0.864
	Hepatobiliary and Pancreatic Surgery	4 (4.7)	2 (2.4)	
	Dermatology	3 (3.5)	2 (2.4)	
	Gynecology	6 (7.0)	9 (10.8)	
	Otolaryngology	3 (3.5)	6 (7.2)	
	Oncology	3 (3.5)	1 (1.2)	
	Gastrointestinal	18 (20.9)	16 (19.3)	
	Pulmonary	18 (20.9)	13 (15.7)	
	Breast	5 (5.8)	4 (4.8)	
	Neurology	0	1 (1.2)	
	Urology	5 (5.8)	4 (4.8)	
Ordered job type, n (%)	Physician	259 (100)	174 (75.0)	<0.001
	Pharmacist	0	58 (25.0)	
Median non compliance period (range), day		41 (15-140)	29 (1-65)	0.021
Compliance rate, n (%)		57 (66.3)	72 (86.7)	0.002

HBV, hepatitis B virus; PBPM, protocol-based pharmacotherapy management

#### UPC-PBPM

There were 634 treatments before PBPM and 585 after initiation of PBPM. Bevacizumab was the most frequently used agent, accounting for approximately 80% of cases. Anti-VEGF therapy was used most frequently for colorectal cancer, accounting for 27.3% of cases before PBPM and 23.4% after PBPM. The rate of compliance with measurement of UPC was 98.7% before PBPM and 99.3% after PBPM; the percentage of laboratory tests ordered by pharmacists increased to 0.9% after introduction of PBPM (Table 3).

**Table 3:** Comparison of rates of compliance with laboratory testing and patient background characteristics before and after introduction of UPC-PBPM

Variable		Pre-PBPM	Post-PBPM	*P*-value
Administrations (n)		634	585	
Male sex, n (%)		343 (54.1)	281 (48.0)	0.039
Median age (range), years		67 (23-93)	66 (37-94)	0.134
Type of cancer, n (%)	Stomach cancer	37 (5.8)	33 (5.6)	0.039
	Hepatocellular carcinoma	105 (16.6)	98 (16.8)	
	Colorectal cancer	173 (27.3)	137 (23.4)	
	Breast cancer	57 (9.0)	50 (8.5)	
	Brain tumor	56 (8.8)	54 (9.2)	
	Lung cancer	101 (15.9)	74 (12.6)	
	Gynecological cancer	105 (16.6)	139 (23.8)	
Chemotherapy, n (%)	Bevacizumab	529 (83.4)	493 (84.3)	0.064
	Ramucirumab	99 (15.6)	92 (15.7)	
	Aflibercept	6 (0.9)	0	
Ordered job type, n (%)	Physician	626 (100)	576 (99.1)	0.026
	Pharmacist	0	5 (0.9)	
	Unmeasured	8	4	
Compliance rate (%)		98.7	99.3	0.583

PBPM, protocol-based pharmacotherapy management; UPC, urine protein/creatinine ratio

#### Mg-PBPM

There were 95 treatments before PBPM and 122 after the start of PBPM. The proportion of patients receiving panitumumab decreased from 73.7% before PBPM to 48.4% after starting PBPM. The prevalence of colorectal cancer was 82.1% in the pre-PBPM period and 71.3% in the PBPM period. The frequency of serum magnesium levels with a Common Terminology Criteria for Adverse Events grade of ≤ 1 was 95.8% in the pre-PBPM period and 95.1% in the PBPM period. The rate of compliance with serum magnesium measurement increased slightly from 95.8% to 99.2% after implementation of Mg-PBPM. After implementation of PBPM, 3.3% of laboratory tests were ordered by pharmacists (Table 4).

**Table 4:** Comparison of rates of compliance with laboratory testing and patient background characteristics before and after introduction of Mg-PBPM

Variable		Pre-PBPM	Post-PBPM	*P*-value
Administrations (n)		95	122	
Male sex, n (%)		36 (37.9)	67 (54.9)	0.014
Median age (range), years		63 (32-75)	63 (45-83)	0.085
Type of cancer, n (%)	Colorectal cancer	78 (82.1)	87 (71.3)	0.078
	Head and neck cancer	17 (17.9)	35 (28.7)	
Chemotherapy, n (%)	Cetuximab	25 (26.3)	63 (51.6)	<0.001
	Panitumumab	70 (73.7)	59 (48.4)	
Serum magnesium CTCAE grade, n (%)	Normal	30 (31.6)	57 (46.7)	0.007
	G1	61 (64.2)	59 (48.4)	
	G2	0	4 (3.3)	
	G3	0	1 (0.8)	
	Unmeasured	4 (4.2)	1 (0.8)	
Ordered job type, n (%)	Physician	91 (100)	117 (96.7)	0.137
	Pharmacist	0	4 (3.3)	
	Unmeasured	4	1	
Compliance rate (%)		95.8	99.2	0.171

CTCAE, Common Terminology Criteria for Adverse Events; Mg, magnesium; PBPM, protocol-based pharmacotherapy management

### Changes in types of pharmacists’ queries after the start of PBPM

**Fig. 2:**
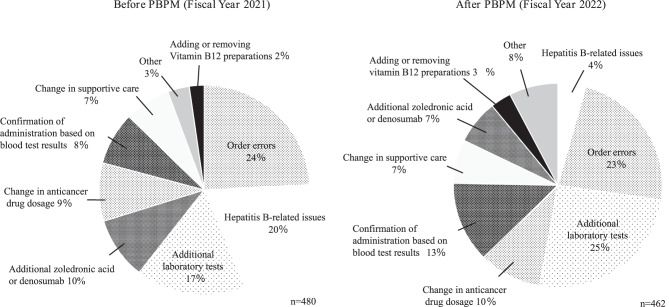
Frequency and nature of prescription queries in the outpatient chemotherapy unit before (FY 2021) and after (FY 2022) the start of PBPM. The total number of prescription queries and percentages for each type of query in the outpatient chemotherapy unit before and after the start of PBPM are shown. FY, fiscal year; PBPM, protocol-based pharmacotherapy management

There were 480 queries from pharmacists to physicians in the 2021 FY, which decreased to 462 in the 2022 FY. After the start of PBPM, the percentage of queries regarding HBV-related prescriptions decreased markedly from 20% to 4% (Fig. 2).

Table 5 shows the details of the pharmacists’ queries regarding Hepatitis B-related issues　and addition of laboratory test items by fiscal year. HBV-PBPM were started in January 2021. In the 2021 fiscal year, there were 94 queries regarding the Hepatitis B-related issues. However, after 2022 fiscal year, when PBPM was fully implemented, queries regarding the Hepatitis B-related issues decreased to about 20. UPC-PBPM and Mg-PBPM were started in August 2022. In the 2022 fiscal year, there were 22 queries regarding the UPC ratio and three queries regarding serum magnesium. However, in the 2023 fiscal year, when PBPM was fully implemented, queries regarding the UPC ratio decreased to 13 and queries regarding serum magnesium decreased to 0. Approximately 85% of pharmacists’ proposals to physicians regarding addition of laboratory test items were accepted. However, it became clear that the number of queries related to ICI therapy has been increasing year by year. Table 6 shows annual changes in the numbers of PBPM strategies implemented by pharmacists. The numbers of PBPM strategies implemented by pharmacists has been increasing over the years, particularly in HBV-PBPM and UPC-PBPM.

**Table 5:** Annual numbers of queries and acceptances for additional test items from pharmacists to physicians after the introduction of three types of PBPM

Hepatitis B-related issues	Fiscal year		
	2021	2022	2023
Total, n (%)	60/94 (63.8)	16/20 (80.0)	15/22 (68.2)
Queries regarding addition of laboratory test items	Fiscal year		
	2021	2022	2023
UPC ratio, n	5/7	20/22	9/13
Serum magnesium, n	3/3	2/3	0
ICI-related laboratory tests, n	19/22	39/45	48/64
Other, n	42/49	42/48	91/105
Total, n (%)	69/81 (85.2)	103/118 (87.3)	148/182 (81.3)

The data show the number of physicians’ acceptances/number of pharmacists’ queries. ICIs, immune checkpoint inhibitors; PBPM, protocol-based pharmacotherapy management; UPC, urinary protein/creatinine

**Table 6:** Annual changes in the numbers of PBPM strategies implemented by pharmacists

Laboratory tests ordered by pharmacists (*n*)	Fiscal year		
	2021	2022	2023
HBV-PBPM	29	188	280
UPC-PBPM	0	11	35
Mg-PBPM	0	8	9
Total	29	207	324

The numbers of PBPM strategies implemented by pharmacists has been increasing over the years, particularly in HBV-PBPM and UPC-PBPM. HBV, hepatitis B virus; PBPM, protocol-based pharmacotherapy management; UPC, urinary protein/creatinine

## Discussion

In this study, we investigated the number of prescription queries from pharmacists to physicians and their acceptance rates in our outpatient chemotherapy unit, and introduced three PBPM strategies that we anticipated would be effective. It was thought that more effective interventions could be made by clarifying the problems at our own facility [8]. Our survey revealed that rates of compliance with HBV-DNA quantification improved significantly after implementation of PBPM. Before the start of PBPM, requests for additional tests were made by telephone, email, or via the medical records, but the compliance rate was low at 66.3%. The compliance rate increased significantly to 86.7% when the pharmacists started PBPM (*p* = 0.002). The median duration of noncompliance decreased significantly from 41 days (range 15–140) in the pre-PBPM period to 29 days (range 1–65) in the PBPM period (*p* = 0.021). Previous studies have reported increases in the implementation rate of HBV-DNA monitoring to 66.7% (*n* = 12) [5], 81.8% (*n* = 44) [6], 93.9% (*n* = 23) [9], 98.3% (*n* = 60) [10], and 100% (*n* = 21) [11] following initiation of PBPM by pharmacists. Our HBV-DNA quantification survey included a larger number of patients than in previous reports, with an adherence rate of 86.7% in 83 patients. Eleven patients did not adhere to the protocol because the pharmacist forgot to order the test; however, the median duration of nonadherence with the test was 29 days post-PBPM, which is shorter than the 41 days pre-PBPM. We believe that pharmacist-driven PBPM minimizes the risk of HBV reactivation in cancer patients and may contribute to safer implementation of chemotherapy.

The American College of Clinical Pharmacy released position statements on collaborative drug therapy management (CDTM) in the United States in 1997 and 2003. Since 2003, CDTM has spread throughout the United States, promoting team-based medical care and reducing the workload of physicians [12]. The CDTM activities implemented include requesting clinical laboratory tests, adjusting the strength of medication, and changing the frequency of administration [13]. In Japan, the Japanese Society of Pharmaceutical Health Care and Sciences released a manual introducing PBPM in 2016 [3] and the Japanese Hospital Pharmacists Association introduced specific practical examples of PBPM in the same year [2], which led to an increase in the number of facilities actively introducing PBPM. PBPM has been reported to be useful in cancer chemotherapy [14–16], when administering high-risk agents [17–19], in patients with human immunodeficiency virus [20] in Japan. While the usefulness of PBPM in patients receiving immunosuppressive therapy or injectable anticancer therapy has been reported [21], the rate of implementation of HBV monitoring in patients receiving oral anticancer agents is reportedly low [10]. Therefore, PBPM should be considered for these patients. While the present study demonstrated the usefulness of PBPM for HBV-DNA monitoring, it could not confirm that PBPM by pharmacists was useful in terms of increasing the rates of compliance with measurement of the UPC ratio during anti-VEGF therapy or the serum magnesium level during anti-EGFR antibody therapy. Between April 1, 2021 and March 31, 2022, queries regarding addition of laboratory test items were the second most frequent type of enquiry after those related to hepatitis B. Therefore, we introduced two PBPMs: UPC-PBPM to determine the feasibility of administering anticancer agents and Mg-PBPM to detect adverse events caused by drug administration at an early stage. However, this study failed to demonstrate their usefulness. In Japan, PBPM has been implemented in many facilities for measurement of the UPC ratio and serum magnesium level, and has been discussed at academic conferences. However, to the best of our knowledge, there are few published reports on this topic. According to the report by Ito et al., the introduction of PBPM increased the rate of urine testing from 74.7% to 99.2% [22]. While PBPM is very effective in terms of increasing measurement rates, at our institution, where the pre-PBPM measurement rate was already high, the usefulness of UPC-PBPM and Mg-PBPM was not demonstrated. The reason for this is that in the case of HBV-DNA quantitative testing, which requires measurement every 3 months, after ordering the necessary test items for each treatment, it is necessary to order HBV-DNA quantitative testing at the required time separately. The UPC ratio and serum magnesium level requires measurement at each treatment, and the frequency of forgetting to measure is low because physicians copy the blood draw order from the previous treatment and re-order it. We introduced UPC-PBPM and Mg-PBPM anticipating that they would be useful, but in reality, the number of tests ordered by pharmacists was low. Therefore, we revisited the content of queries regarding test items before and after introducing UPC-PBPM and Mg-PBPM. Queries regarding the UPC ratio and serum magnesium before and after introduction of PBPM were 12.3% (10/81 cases) in the 2021 fiscal year and 21.2% (25/118 cases) in the 2022 fiscal year, while queries regarding the addition of laboratory test items related to ICIs (including thyroid-stimulating hormone, FT4, FT3, KL-6, and urine tests) were 27.2% (22/81) and 38.1% (45/118), respectively. These findings show that queries regarding the addition of laboratory test items related to ICIs have been increasing annually. Previous research has demonstrated the usefulness of implementing PBPM for testing when ICIs are used [14], and the findings at our hospital also suggest the need to initiate PBPM in the future. Furthermore, despite the initiation of PBPM, 25 (21.2%) of the 118 queries in the 2022 FY and 13 (7.1%) of the 182 queries in 2023 FY concerned measurement of the UPC ratio or serum magnesium level, and there are approximately 20 Hepatitis B-related issues queries per year suggesting that physicians may not have delegated ordering authority to pharmacists. Physicians at our hospital delegate ordering authority to pharmacists but must operate the electronic medical records system themselves to establish the necessary settings. This process is cumbersome and may hinder the delegation of authority. Furthermore, Alhossan et al. identified a lack of knowledge among other healthcare professionals regarding PBPM [23]. The knowledge that PBPM can positively influence outcomes related to pharmacotherapy should be shared with other healthcare professionals. While the implementation of PBPM by pharmacists has little financial impact on hospitals, it can increase the value of pharmacists and contribute to team-based care [24].

### Limitations

In this study, there was a difference in the start date for HBV-PBPM (January 2021) and UPC-PBPM/Mg-PBPM (August 2022) because of the desire to initiate chemotherapy promptly but safely. Therefore, the content of queries in the 2021 fiscal year (April 1, 2021 to March 31, 2022) and the 2022 fiscal year (April 1, 2022 to March 31, 2023) include data from both before and after the start of PBPM. However, it is important to note that the data for the 2023 fiscal year (April 1, 2023 to March 31, 2024) shows the content of queries when PBPM was fully implemented.

## Conclusion

The introduction of PBPM improves compliance with the laboratory test items necessary during chemotherapy for cancer patients and enables the delivery of safe treatment.

## Data Availability

All the data generated or analyzed during this study are included in this article.

## Abbreviations

ACCP: American College of Clinical Pharmacy
CDTM: collaborative drug therapy management
EGFR: epidermal growth factor receptor
HBV: hepatitis B virus
ICIs: immune checkpoint inhibitors
PBPM: protocol-based pharmacotherapy management
UPC: urine protein/creatinine
VEGF: vascular endothelial growth factor

## References

[1] Ministry of Health. Labour and welfare. Promotion of team medical care through collaboration with medical staffs. 2010. Available from: https://www.mhlw.go.jp/shingi/2010/05/dl/s0512-6h.pdf.

[2] Japanese Society of Hospital Pharmacists. How to facilitate protocol-based pharmacotherapy management (PBPM) and specific practical examples (Ver.1.0). 2016. Available from: https://www.jshp.or.jp/activity/guideline/20160331-1.pdf.

[3] Japanese society of pharmaceutical health care and sciences. Protocol-based pharmacotherapy management (PBPM) implementation manual. 2016. Available from: https://www.jsphcs.jp/wp-content/uploads/2024/10/20160613-1.pdf.

[4] The Japan Society of Hepatology. Hepatitis B Treatment Guidelines. 2022. Available from: https://www.jsh.or.jp/lib/files/medical/guidelines/jsh_guidlines/B_v4.pdf.

[5] Akira T, Ryoo T, Yasuhiro S, Seiichi M, Yuji W, Tomoyuki O, et al. Evaluation of the implementation of HBV-related marker testing during cancer chemotherapy and the effectiveness of medical safety measures. J Educ Chang Jpn Soc Of Pharm Oncol. 2017;6:16–23. Available from: https://jaspo-oncology.org/file/224#page=18.

[6] Chieko S, Masaaki I, Takao O, Naoyuki U. Construction of protocol-based pharmacotherapy management (PBPM) using an electronic medical record interlocking support system to prevent hepatitis B virus reactivation induced by cancer chemotherapy. Jpn J Pharm Health Care Sci. 2022;48(1):9–19. Available from: https://www.jstage.jst.go.jp/article/jjphcs/48/1/48_9/_pdf/-char/ja

[7] Kanda Y. Investigation of the freely available easy-to-use software ‘EZR’ for medical statistics. Bone Marrow Transpl. 2013;48(3):452–58. Available from: https://pmc.ncbi.nlm.nih.gov/articles/PMC3590441/10.1038/bmt.2012.244 PMC3590441 23208313

[8] Sato M, Fujita S, Kimura M, Takeuchi K, Hamahata Y, Matsuda Y. Designing effective protocol-based pharmacotherapy management: assessment of the development processes and outcomes in inflammatory bowel disease care prescription management. Pharm (basel). 2025;13(1). Available from: https://pmc.ncbi.nlm.nih.gov/articles/PMC11860031/10.3390/pharmacy13010017 PMC11860031 39998015

[9] Daisuke M, Hiroki T, Kensaku Y, Toru M, Daiki Y, Yukiko A, et al. Pharmacist orderings for blood and urine tests related to pharmaceuticals -practice of protocol-based pharmacotherapy management (PBPM)-. Jpn J Pharm Health Care Sci. 2021;47(7):345–57. Available from: https://www.jstage.jst.go.jp/article/jjphcs/47/7/47_345/_pdf/-char/ja

[10] Keiko F, Hideki K, Akane N, Masahiro O, Eguchi Y, et al. The department of pharmacy at Fukuoka University Hospital’s role in preventing hepatitis B virus reactivation in patients taking oral anticancer drugs. Gan To Kagaku Ryoho. 2023;50(8):885–89. 37608414

[11] Hiroyuki W, Ikuko H, Yuka N, Noriyuki H, Tatsuichi A, Takashi Y. Evaluation of effectiveness and implementation of protocol-based pharmacotherapy management for prevention of hepatitis B virus reactivation induced cancer chemotherapy. J Educ Chang Jpn Soc of Pharm Oncol. 2016;5:13–18. Available from: https://jaspo-oncology.org/file/225#page=15.

[12] McBane SE, Dopp AL, Abe A, Benavides S, Chester EA, Dixon DL, et al. Collaborative drug therapy management and comprehensive medication management-2015. Pharmacotherapy. 2015;35(4):e39–50. Available from: https://accpjournals.onlinelibrary.wiley.com/doi/epdf/10.1002/phar.1563 10.1002/phar.1563 25884536

[13] Mishra P, Thomas J. Survey of collaborative drug therapy management in U.S. hospitals. Am J Health Syst Pharm. 2017;74(21):1791–905. Available from: 10.2146/ajhp151058 10.2146/ajhp151058 29070500

[14] Ikesue H, Kusuda K, Satsuma Y, Nishiwaki F, Miura R, Masuda Y, et al. Evaluation of the usefulness of protocol-based pharmacist-facilitated laboratory monitoring to ensure the safety of immune checkpoint inhibitors in patients with lung cancer. J Clin Pharm Ther. 2020;45(6):1288–94. Available from: https://pmc.ncbi.nlm.nih.gov/articles/PMC7687122/pdf/JCPT-45-1288.pdf 10.1111/jcpt.13207 PMC7687122 32519774

[15] Nakamura N, Shiraiwa H, Haruna Y, Ichijima T, Takeda T, Hasegawa K, et al. Effectiveness of protocol-based pharmacotherapy management collaboration between hospital and community pharmacists to address capecitabine-related hand-foot syndrome in cancer patients: a retrospective study. J Pharm Health Care Sci. 2021;7(1):8. Available from: https://pmc.ncbi.nlm.nih.gov/articles/PMC7919314/pdf/40780_2021_Article_191.pdf doi: 10.1186/s40780-021-00191-1. PMC7919314 33641672

[16] Yanagawa T, Kataoka K, Yamashita N, Yanai M, Tanaka K, Bando J, et al. Effects of introducing PBPM for outpatient cancer drug therapy and its impact on Physician workload. Gan To Kagaku Ryoho. 2024;51(7):747–51. Available from: https://www.pieronline.jp/content/article/0385-0684/51070/747. 39191693

[17] Asai Y, Nakano Y, Yanagawa T, Takahashi M, Iwamoto T. Impact of a collaborative pharmacist-cardiovascular Surgeon protocol for high risk of postoperative delirium on benzodiazepine prescription trends in hospitalized patients. Biol Pharm Bull. 2025;48(2):177–83. Available from: https://www.jstage.jst.go.jp/article/bpb/48/2/48_b24-00708/_pdf/-char/en. 10.1248/bpb.b24-00708 40024718

[18] Asai Y, Yanagawa T, Takahashi M. Effect of pharmacist-led intervention protocol on preventing postoperative delirium after elective cardiovascular surgery. PLoS One. 2023;18(10):e0292786. Available from: https://pmc.ncbi.nlm.nih.gov/articles/PMC10569577/pdf/pone.0292786.pdf 10.1371/journal.pone.0292786 PMC10569577 37824500

[19] Katada Y, Nakagawa S, Minakata K, Odaka M, Taue H, Sato Y, et al. Efficacy of protocol-based pharmacotherapy management on anticoagulation with warfarin for patients with cardiovascular surgery. J Clin Pharm Ther. 2017;42(5):591–97. Available from: https://onlinelibrary.wiley.com/doi/epdf/10.1111/jcpt.12560 10.1111/jcpt.12560 28503837

[20] Urano K, Ishibashi M, Matsumoto T, Ohishi K, Muraki Y, Iwamoto T, et al. Impact of physician-pharmacist collaborative protocol-based pharmacotherapy management for HIV outpatients: a retrospective cohort study. J Pharm Health Care Sci. 2020;6:9. Available from: https://pmc.ncbi.nlm.nih.gov/articles/PMC7193403/pdf/40780_2020_Article_165.pdf 10.1186/s40780-020-00165-9 PMC7193403 32377369

[21] Komuro M, Toda Y, Ishikawa S, Hyakutake H, Watanabe T, Shimanuki Y, et al. Operational construction and effectiveness verification of an HBV reactivation management protocol for cancer chemotherapy patients based on medical information system safety management. Gan To Kagaku Ryoho. 2025;52(6):457–61. Available from: https://www.pieronline.jp/content/article/0385-0684/52060/457. 40563152

[22] Minori I, Yasuhiko S, Keiji S, Shinichi K, Kouichi H. Approach for appropriate use of urinary protein related anti-VEGF antibody by introducing the PBPM. J Jpn Soc Hosp Pharm. 2017;53(6):703–07. Available from: https://mol.medicalonline.jp/archive/search?jo=dg4hppha%26vo=53%26issue=6

[23] Alhossan A, Alazba A. Barriers interfering with establishment of collaborative drug therapy management (CDTM) agreements between clinical pharmacists and physicians. Saudi Pharm J. 2019;27(5):713–16. Available from: https://pmc.ncbi.nlm.nih.gov/articles/PMC6598211/pdf/main.pdf 10.1016/j.jsps.2019.04.006 PMC6598211 31297026

[24] Thomas J, Bharmal M, Lin SW, Punekar Y. Survey of pharmacist collaborative drug therapy management in hospitals. Am J Health Syst Pharm. 2006;63(24):2489–99. Available from: https://academic.oup.com/ajhp/article-abstract/63/24/2489/5134611?redirectedFrom=fulltext%26login=true 10.2146/ajhp050205 17158697

